# Identification of Iroquois Fire Victims

**Published:** 1904-03-15

**Authors:** Geo. W. Cook


### IDENTIFICATION OF IROQUOIS FIRE VICTIMS.

BY GEO. W. COOK, D.D.S.

I was called on New Year's day to assist a bereaved husband to identify his wife, who had been my patient something like a year previous, and in whose mouth I had inserted a bridge, which I felt sure I could positively identify.

The relatives had found several bodies, one of which they were almost certain was the one sought. To these they directed my attention, but on thorough examination neither of them proved the right one. I then made a systematic examination of the bodies there and the ninety-second proved to be the body of the wife of my friend.

One can scarcely imagine a more difficult task than that of opening the mouths of the dead, especially in these cases, as they were burned until the flesh would in most instances readily leave the bone. Another difficult matter was that there were so many women, and, as a rule, they differ little in general size and characteristics.

When we consider that on the morning of the third day there were still over two hundred unidentified and seventy-two percent of these had more or less dental work in the way of fillings, plates and bridge-work, it would have been extremely difficult to have identified all the victims of this disaster had it not been for these dental operations. We believe at the present writing all but one was positively identified.

It seems at the present enlightened day the public press should call attention to such means as dental operations for the identification of individuals, where the features and general appearance had been so mutilated at to render it impossible to recognize by ordinary means.

It seems that this would also illustrate the importance of dentists keeping a full record of all their operations; for instance, there was one woman who had an upper artificial denture, in which gold filling had been inserted, and this individual was among the last to be recognized. This was done largely by exclusion. The friends accepted the body after all the rest that had answered the general description had been claimed.

Many persons had to be recognized by various means that were more or less uncertain, such for instance as scars from appendicitis operations, corn plasters on the feet, all of which might appear on the bodies of many individuals. —*American Journal.*

